# Cognitive testing in the COVID-19 era: can existing screeners be adapted for telephone use?

**DOI:** 10.2217/nmt-2020-0040

**Published:** 2020-11-10

**Authors:** Andrew J Larner

**Affiliations:** ^1^Cognitive Function Clinic, Walton Centre for Neurology & Neurosurgery, Lower Lane, Fazakerley, Liverpool L9 7LJ, UK

**Keywords:** COVID-19, dementia, mild cognitive impairment, telediagnosis

## Abstract

**Aim:** To examine whether two existing cognitive screeners might be adapted for telephone administration by omission of item content requiring visual cues or assessment. **Materials & methods:** Data from a test accuracy study of Mini-Addenbrooke’s Cognitive Examination (MACE) and Free-Cog were used to derive scores for ‘Tele-MACE’ and ‘Tele-Free-Cog’. **Results:** As in the index study, both Tele-MACE and Tele-Free-Cog proved sensitive for dementia diagnosis. Tele-MACE had a better balance of sensitivity and specificity than observed with MACE. Tele-MACE was sensitive for mild cognitive impairment diagnosis, whereas Tele-Free-Cog was more specific for mild cognitive impairment. **Conclusion:** Existing cognitive screeners may be adapted for telephone administration. Such developments may prove necessary in the COVID-19 era as remote rather than face-to-face cognitive assessment increasingly becomes the established norm.

**Practice points**

- Because of COVID-19, cognitive screening will increasingly be undertaken remotely, by videolink or telephone, rather than face-to-face.
- Cognitive screening instruments suitable for administration by telephone may be increasingly required, necessitating omission of material requiring visual cues or assessment.
- Although some instruments designed specifically and validated for telephone use are available, none has been identified as optimal by systematic reviews, and hence development of others for future use may be appropriate.
- Adaptations of the 30-point Mini-Addenbrooke’s Cognitive Examination (MACE) and Free-Cog, omitting material inappropriate for telephone administration or in the patient’s home, reduced test denominators from 30 to 25 and 21 respectively.
- Tele-MACE and Tele-Free-Cog scores derived from a previous pragmatic test accuracy study showed good sensitivity for dementia diagnosis and Tele-MACE was also sensitive for mild cognitive impairment diagnosis (all ≥ 0.8).
- Existing cognitive screeners may be adapted for telephone administration.

In the COVID-19 (SARS-Cov-2) era, with the requirement for social distancing, face-to-face (F2F) hospital-based clinical consultations have been largely replaced by distance, remote or teleconsultations, conducted by videolink or by telephone in the patient’s own home [1]. This ‘new normal’ poses challenges for the administration of cognitive screening instruments to patients with complaints of poor or declining memory. Some guidance on this issue has been provided by the Royal College of Psychiatrists [2].

Although many of the available cognitive screening instruments might potentially be administered via videolink in the same manner as F2F, there are some potential problems with the use of this technology. It may be unavailable or unfamiliar to the demographic most likely to develop cognitive impairment. Tests of visuoperceptual function (e.g., fragmented letters or pictures), visuomotor skills (e.g., drawing intersecting pentagons, Necker cube) or a combination (e.g., clock drawing) may be difficult to perform, dependent on the quality of the videolink, and patients would have to provide their own pen and paper. Hence, testing patients’ cognitive function in their own homes by telephone may become increasingly the standard practice.

Fortunately, some cognitive instruments designed specifically for telephone use are available, such as the Telephone Interview for Cognitive Status (TICS) [3]. Another approach has been to adapt existing cognitive screeners by omitting those items which cannot be administered by telephone. There are several such adaptations of the Mini-Mental State Examination [4] and of the Montreal Cognitive Assessment [5,6]. Attempts to administer the Test Your Memory test by telephone have also been reported [7]. Other screeners are already suitable (‘oven-ready’) for telephone use since they eschew tests that require visual cues or visual assessment, for example, the Six-item Cognitive Impairment Test [8]. There is overlap here with the development of instruments designed for use with visually impaired patients, which might also be suitable for administration by telephone.

The Cochrane Dementia and Cognitive Improvement Group is currently undertaking a systematic review of the diagnostic test accuracy of remote multidomain cognitive assessment (telephone and video call) for dementia (TJ Quinn, Personal Communication). Existing systematic reviews have confirmed that telephone assessment of cognitive function is a promising approach but have not identified an optimal instrument for this medium [9,10]. Hence development of further telephone screeners, based on existing cognitive screening tests, may be of value as applicable to future practice. To the author’s knowledge, no telephone adaptation of any of the iterations of the Addenbrooke’s Cognitive Examination has been described. The aim of this study was to examine the potential utility of an adaptation of the Mini-Addenbrooke’s Cognitive Examination (MACE) [11] omitting all visually-based items and hence suitable for telephone use, and likewise for another brief cognitive screener, Free-Cog.

## Materials & methods

The dataset of a previously reported prospective pragmatic test accuracy study of two cognitive screeners, MACE and Free-Cog, was used [12]. In this study, data were collected from consecutive new patient referrals to a dedicated cognitive disorders clinic over a 12-month period (November 2017–October 2018 inclusive). There were no specific inclusion/exclusion criteria other than exclusion for a pre-existing diagnosis of dementia. Study protocol was approved by the local institutional committee on human research and subjects gave informed consent.

MACE and Free-Cog differ in the number of subdomains examined (5 vs 12; Table 1) but have the same score range (0–30, impaired to normal) and take similar time to perform (around 10 min). MACE has cutoffs specified in the index paper (≤25/30 and ≤21/30) [11], and a cutoff of ≤22/30 was empirically determined for Free-Cog [12].

**Table 1.** Item content of MACE and Free-Cog and their adaptations for telephone use.

	MACE	‘Tele-MACE’	Free-Cog	‘Tele-Free-Cog’
			**‘Cognitive function’**	
General knowledge	–	–	1	1
Orientation: time	4	4	3	3
Orientation: place	–	–	3	Omitted
Registration	7 (7-item name and address, scored on third presentation)	7	0 (5 words)	0
Calculation	–	–	3	3
Attention/concentration	–	–	2	2
Memory recall:	7 (Recall of previously presented 7-item name and address)	7	5 (Recall of previously presented 5 words)	5
Verbal fluency in 1 minute	7 (phonemic: P words)	7	1 (semantic: animals)	1
Language: naming	–	–	2	Omitted
Language: repetition	–	–	1	1
Language: write a sentence	–	–	1	Omitted
Visuospatial abilities: clock drawing test	5	Omitted	3	Omitted
			**‘Executive function’**	
	–	–	5 (questions relating to social function, travel, home, emergency and self-care function)	5
**Total score**	**30**	**25**	**30**	**21**

MACE: Mini-Addenbrooke’s Cognitive Examination.

Each test was adapted to be suitable for telephone use by omitting item content which was either inappropriate to home testing (e.g., orientation in place) or could not be administered by telephone (e.g., any test requiring visual cues or assessment). In consequence, test denominators were reduced to 25 for ‘Tele-MACE’ and to 21 for ‘Tele-Free-Cog’, the latter solely affecting the ‘cognitive function’ component (denominator reduced from 25 to 16) but with preservation (denominator 5) of the ‘executive function’ component (Table 1).

Revised test scores were cross-classified with reference diagnoses from the original study. Therein, standard diagnostic criteria for dementia (DSM-IV) and mild cognitive impairment (MCI; Petersen [13]) were used, these older criteria having been the standard practice of the clinic for many years and retained to permit comparison with tests previously assessed despite the more recent publication of revised criteria (e.g., DSM-5). Criterion diagnosis of dementia, MCI or subjective memory complaint (SMC), was by judgment of an experienced clinician based on diagnostic criteria but did not use either MACE or Free-Cog score in order to avoid review bias. Analyses were conducted comparing patients with dementia versus no dementia (combined MCI and SMC) and comparing patients with MCI versus SMC.

Using the revised test scores, receiver operating characteristic curves were constructed in order to define optimal test cutoffs using the maximal Youden index. At this optimal cutoff, standard summary measures of discrimination were calculated (sensitivity, specificity, positive and negative predictive values) with 95% CI.

## Results

For all 141 patients initially assessed with both MACE and Free-Cog (F:M = 61:80, 43% female; age range 28 to 88 years, median 62 years) data were available for all individual test items to permit Tele-MACE and Tele-Free-Cog scores to be derived.

Reference diagnoses were: dementia 15 (Alzheimer’s disease/vascular dementia/mixed dementia 10, frontotemporal dementia 2, alcohol-related dementia 2, dementia with Lewy bodies 1); MCI 45 (amnestic 22, single nonmemory domain 1, multiple domain 22); and SMC 81. The patients diagnosed with dementia were significantly older than those without dementia, and those with MCI were significantly older than those with SMC. No significant difference with respect to gender was found (Table 2) [12].

**Table 2.** Patient diagnostic groups by age, gender and test scores.

	Total	Dementia	MCI	SMC	p-value
n	141	15	45	81	–
Prevalence	–	0.11	0.32	0.57	–
Age (mean +/- SD)	61.8 +/-13.2	71.3 +/-9.0	69.8 +/-10.1	55.6 +/-11.9	Dementia vs MCI + SMC: p < 0.01 MCI vs SMC: p < 0.001
F:M (% female)	61:80 (43)	5:10 (33)	23:22 (51)	33:48 (41)	p > 0.1
Tele-MACE score (0–25) (mean +/- SD)	16.2 +/-5.2	9.5 +/-3.6	14.8 +/-4.7	18.3 +/-4.4	Dementia vs MCI + SMC: p < 0.001 MCI vs SMC: p < 0.001
Tele-Free-Cog score (0–21) (mean +/- SD)	14.8 +/-4.4	8.0 +/-3.4	13.6 +/-3.8	16.7 +/-3.2	Dementia vs MCI + SMC: p < 0.001 MCI vs SMC: p < 0.001

MCI: Mild cognitive impairment; SD: Standard deviation; SMC: Subjective memory complaint.

As anticipated, there was a high positive correlation between scores on MACE and Tele-MACE (0.987) and between Free-Cog and Tele-Free-Cog scores (0.976).

Mean scores for Tele-MACE and Tele-Free-Cog differed significantly between patients diagnosed with dementia compared with those without dementia, and between those with MCI compared with those with SMC (Table 2, bottom two rows).

From the receiver operating characteristic curves (not shown), maximal Youden index for Tele-MACE was found at cutoff ≤12/25 for diagnosis of dementia versus no dementia and at ≤19/25 for diagnosis of MCI versus SMC. At these defined optimal cutoffs, Tele-MACE was both sensitive and specific for dementia diagnosis and sensitive for MCI diagnosis (all ≥0.8) with high NPV for both diagnoses (>0.85; Table 3).

**Table 3.** Measures of discrimination (with 95% CI) for diagnosis of dementia versus no dementia and mild cognitive impairment versus subjective memory complaint using Tele-MACE and Tele-Free-Cog at optimal cutoffs defined by maximal Youden index.

	Dementia vs no dementia (=MCI + SMC)		MCI vs SMC	
n	141		126	
Test cutoff	Tele-MACE ≤12/25	Tele-Free-Cog ≤10/21	Tele-MACE ≤19/25	Tele-Free-Cog ≤13/21
Sensitivity (Sens)	0.87 (0.69–1.00)	0.80 (0.60–1.00)	0.84 (0.74–0.95)	0.56 (0.41–0.70)
Specificity (Spec)	0.80 (0.73–0.87)	0.89 (0.83–0.94)	0.52 (0.41–0.63)	0.84 (0.76–0.92)
Youden index (Sens + Spec – 1)	0.67	0.69	0.36	0.40
PPV	0.34 (0.19–0.49)	0.46 (0.27–0.65)	0.49 (0.38–0.61)	0.66 (0.51–0.81)
NPV	0.98 (0.95–1.00)	0.97 (0.94–1.00)	0.86 (0.76–0.96)	0.77 (0.69–0.86)
Accuracy (Acc)	0.81 (0.74–0.87)	0.88 (0.83–0.93)	0.63 (0.55–0.72)	0.74 (0.66–0.81)

MACE: Mini-Addenbrooke’s Cognitive Examination; MCI: Mild cognitive impairment; NPV: Negative predictive value; PPV: Positive predictive value; SMC: Subjective memory complaint.

Maximal Youden index for Tele-Free-Cog was found at ≤10/21 for diagnosis of dementia versus no dementia and at ≤13/21 for diagnosis of MCI versus SMC. At these defined optimal cutoffs, Tele-Free-Cog was both sensitive and specific for dementia diagnosis and specific for MCI diagnosis (all ≥ 0.8) but not sensitive for MCI diagnosis (0.52; Table 3).

## Discussion

The pattern of results using these adaptations of existing cognitive screening instruments to make them suitable for telephone administration was similar to that seen in the original study of full MACE and Free-Cog [12], but with some differences.

MACE was very sensitive but not very specific for dementia and MCI diagnoses, whereas Tele-MACE achieved a better balance, with improved specificity, Youden index and correct classification accuracy at the optimal cutoff while retaining acceptably high sensitivity. It would seem that the clock drawing test, the one MACE item omitted in the Tele-MACE (Table 1), may add little to sensitivity while compromising specificity. Most patients in this cohort were at ceiling on the clock drawing item [12], suggesting this test may be too easy (as previously noted for patients with functional cognitive disorder [14]), and only one patient with cognitive impairment had an identical score on MACE and tele-MACE [15].

Like MACE, Free-Cog was very sensitive for dementia diagnosis in the index study, but less so for identification of MCI [12], a pattern recapitulated by Tele-Free-Cog, although reasonable specificity for MCI diagnosis was retained. Both Tele-MACE and Tele-Free-Cog had high NPV for dementia diagnosis, the high sensitivity indicating a low false negative rate, hence a negative result may rule the diagnosis out.

The study has various methodological limitations and shortcomings. First, this is not and does not claim to be a validation study. To validate a new formulation of a cognitive screening test, one would ideally design a study comparing the putative telephone version with F2F testing in a large cohort of patients [10]. However, the extraordinary circumstances of the COVID-19 era may necessitate more flexible, streamlined, approaches to test development and assessment. Second, deriving test scores retrospectively from lengthier tests has the potential to introduce bias, although this approach has been adopted in previous studies and systematic reviews of various cognitive screeners [16,17]. No comment can therefore be made on the acceptability or otherwise of these new telephone formulations to patients. Telephone administration of these instruments might be affected by various issues, such as poor line connection, impaired patient hearing and loss of visual cues apparent during F2F administration. Third, the sample size of the study was relatively small, and no power calculation to estimate sample sizes was undertaken as the study was retrospective, although the sample size fell within the normative ranges calculated as acceptable (25–400) for common research designs [18]. Fourth, another approach, based on experience with stroke patients in whom cognitive testing may be incomplete or only partially complete because of physical and other impairments, might be to retain the same test denominators by imputing missing item scores using explicit rules for completion [19].

Finally, it was not the purpose of the study to examine the important issues of how a diagnosis of dementia or MCI should be established or communicated by telephone.

## Conclusion

Notwithstanding these various issues, the data presented here suggest that simple adaptations of MACE and Free-Cog for telephone use may achieve the high sensitivity desirable for screening instruments and therefore indicate which patients with cognitive symptoms need more in-depth assessment including F2F consultation and hospital-based investigations.
